# Spatial Proteomic Analysis of Isogenic Metastatic Colorectal Cancer Cells Reveals Key Dysregulated Proteins Associated with Lymph Node, Liver, and Lung Metastasis

**DOI:** 10.3390/cells11030447

**Published:** 2022-01-27

**Authors:** Guillermo Solís-Fernández, Ana Montero-Calle, Javier Martínez-Useros, Álvaro López-Janeiro, Vivian de los Ríos, Rodrigo Sanz, Jana Dziakova, Elena Milagrosa, María Jesús Fernández-Aceñero, Alberto Peláez-García, José Ignacio Casal, Johan Hofkens, Susana Rocha, Rodrigo Barderas

**Affiliations:** 1 Molecular Imaging and Photonics Division, Chemistry Department, Faculty of Sciences, KU Leuven, Celestijnenlaan 200F, 3001 Leuven, Belgium; guillermo.solisfernandez@kuleuven.be (G.S.-F.); johan.hofkens@kuleuven.be (J.H.); susana.rocha@kuleuven.be (S.R.); 2 Chronic Disease Programme, UFIEC, Instituto de Salud Carlos III, 28220 Madrid, Spain; ana.monteroc@isciii.es; 3 Translational Oncology Division, OncoHealth Institute, Health Research Institute—Fundacion Jimenez Diaz University Hospital, 28040 Madrid, Spain; javier.museros@quironsalud.es; 4 Molecular Pathology and Therapeutic Targets Group, La Paz University Hospital (IdiPAZ), 28046 Madrid, Spain; aljaneiro@salud.madrid.org (Á.L.-J.); alberto.pelaez@idipaz.es (A.P.-G.); 5 Proteomics Facility, Centro de Investigaciones Biológicas (CIB-CSIC), 28039 Madrid, Spain; vrios@cib.csic.es; 6 Hospital Clínico San Carlos, IdISSC, 28040 Madrid, Spain; elrodriusa@hotmail.com (R.S.); janadziakova@gmail.com (J.D.); elenamilagrosa.molina@salud.madrid.org (E.M.); jmariajesus.fernandez@salud.madrid.org (M.J.F.-A.); 7 Centro de Investigaciones Biológicas (CIB-CSIC), Department of Molecular Biomedicine, 28039 Madrid, Spain; icasal@cib.csic.es

**Keywords:** colorectal cancer, metastasis, spatial proteomics, quantitative proteomics, TMT, mass-spectrometry

## Abstract

Metastasis is the primary cause of colorectal cancer (CRC) death. The liver and lung, besides adjacent lymph nodes, are the most common sites of metastasis. Here, we aimed to study the lymph nodes, liver, and lung CRC metastasis by quantitative spatial proteomics analysis using CRC cell-based models that recapitulate these metastases. The isogenic KM12 cell system composed of the non-metastatic KM12C cells, liver metastatic KM12SM cells, and liver and lung metastatic KM12L4a cells, and the isogenic non-metastatic SW480 and lymph nodes metastatic SW620 cells, were used. Cells were fractionated to study by proteomics five subcellular fractions corresponding to cytoplasm, membrane, nucleus, chromatin-bound proteins, and cytoskeletal proteins, and the secretome. Trypsin digested extracts were labeled with TMT 11-plex and fractionated prior to proteomics analysis on a Q Exactive. We provide data on protein abundance and localization of 4710 proteins in their different subcellular fractions, depicting dysregulation of proteins in abundance and/or localization in the most common sites of CRC metastasis. After bioinformatics, alterations in abundance and localization for selected proteins from diverse subcellular localizations were validated via WB, IF, IHC, and ELISA using CRC cells, patient tissues, and plasma samples. Results supported the relevance of the proteomics results in an actual CRC scenario. It was particularly relevant that the measurement of GLG1 in plasma showed diagnostic ability of advanced stages of the disease, and that the mislocalization of MUC5AC and BAIAP2 in the nucleus and membrane, respectively, was significantly associated with poor prognosis of CRC patients. Our results demonstrate that the analysis of cell extracts dilutes protein alterations in abundance in specific localizations that might only be observed studying specific subcellular fractions, as here observed for BAIAP2, GLG1, PHYHIPL, TNFRSF10A, or CDKN2AIP, which are interesting proteins that should be further analyzed in CRC metastasis.

## 1. Introduction

Colorectal cancer (CRC), with an estimated overall incidence in the general population of 447 per 100,000, is the second most common cancer in Europe after lung cancer [1,2]. Despite screening programs, CRC is diagnosed at advanced metastatic stages in nearby 20–30% of patients, and relapse occurs in about 40–50% of those patients diagnosed at early cancer stages [3]. In the last decade, the use of different schedules of chemotherapies combined with targeted biological therapies has considerably improved the median overall survival for patients with metastatic CRC [1]. Nevertheless, the majority of patients with metastatic CRC progress in spite of their initial treatment receiving second and third line treatments, which results in a less than 10% 5-year survival probability.

For CRC, besides adjacent lymph node colonization, the most common sites of metastasis are the liver (~70%) and lung (~39%); the other less common metastases sites being peritoneum, bone, and nervous system metastases [4]. Liver metastases are more frequently solitary (~46% of CRC patients), while lung metastases often occur together with liver ones (~68%) [4]. Recent progress in metastasis research has expanded our understanding of the molecular and cellular mechanisms behind this process [1,5]; however, it is still far from being fully understood. Metastasis is a multifaceted process comprised of multiple events. The formation of a secondary tumor after colonization at a distant site is preceded by basement membrane invasion and cell migration. Subsequently, cancer cells intravasate into the surrounding vasculature or lymphatic system. Cancer cells that are able to survive in the circulation will be able to reach a secondary tissue by extravasation and colonization [6]. All of these events where cancer cells need to interact with different microenvironments possess unique molecular characteristics [7,8]. There, cancer cells must adapt to each situation, altering the expression, localization, and activation of proteins to generate a metastasis.

Quantitative proteomic analysis of cell lines with the same genetic backgrounds, but differing in their metastatic capabilities, has an immense potential to unveil clinically relevant underlying mechanisms [9,10,11,12]. The most widely used CRC cell models of metastasis are the KM12 cell system and the SW480/SW620 pair of cell lines. The KM12 cell system includes three isogenic cell lines with different metastatic properties derived from a CRC patient in stage II and successive passages in nude mice: non- or poorly-metastatic KM12C cells, liver metastatic KM12SM cells, and liver and lung metastatic KM12L4a cells [13,14]. Numerous studies support a good correlation between the findings observed using the KM12C and KM12SM cells and patient samples, indicating that these isogenic cell lines recapitulate quite effectively critical issues in CRC liver metastasis [15,16,17,18,19]. On the other hand, the SW480/SW620 pair represents the chromosomal instability subtype of CRC that is clinically commonly observed [8,9]. These cells were derived from a primary adenocarcinoma and a lymph node metastasis, respectively [8]. They possess differences in xenograft metastatic potential in vivo [10], migratory propensity, and drug sensitivity [11], which also recapitulates the behavior observed in vivo. 

A key defining feature of eukaryotic cells is precisely their high level of compartmentalization, which is crucial for the portioning of biological processes. Such compartmentalization allows for localizing specific proteins to different locations and for creating distinct chemical environments. Hence, control over protein subcellular localization is a central part of cell physiology [20,21]. Indeed, many biological cell processes, such as signaling cascades, involve changes in protein subcellular localization, and protein mislocalization is often linked to disease [22,23]. In the context of cancer, changes in the abundance or subcellular localization of proteins, such as tumor suppressor or oncoproteins, has been frequently reported [22]. Mislocalization of these proteins can prevent them from carrying on their function, subsequently altering their ability to suppress tumor cells or, for oncoproteins, increase their potential for inducing cancer development, metastasis or drug resistance. Consequently, identification of mislocalized proteins can be of special relevance for the discovery of new cancer diagnostic markers or therapeutic targets.

Previous studies have separately compared the KM12 cell model and the SW480/SW620 cell pair at the proteome and secretome levels [12,13], or focused on the pair KM12C and KM12SM of the KM12 cell system to spatially delineate proteins associated with liver metastasis [24]. However, there has been no report applying quantitative proteomics to analyze jointly the different metastatic properties to lymph nodes (SW480/SW620 cell pair), to the liver, and to the liver and lung (KM12C/KM12SM/KM12L4a cells) to identify proteins associated with CRC metastasis or proteins specific of lymph nodes, liver or lung metastatic niches. Here, we compared the multidimensional protein content of five subcellular fractions (cytoplasmatic proteins, membrane proteins, nuclear proteins, chromatin bound proteins, and cytoskeletal proteins) and the secretome of the three KM12 cell lines and the SW480/SW620 pair of cells by TMT 11-plex quantitative proteomics. The aim of the study consisted of identifying altered proteins in abundance and localization in the most important metastatic niches of CRC besides lymph nodes (liver and lung), to gain further insights into metastatic CRC and to try to find novel relevant proteins associated with the disease, which might serve as diagnostic markers and/or therapeutic targets of intervention. Proteins were measured in parallel for the six separate subcellular fractions, outlining metastasis-associated and tropism-associated proteins. After bioinformatics, alterations in abundance and localization for selected proteins from diverse subcellular localizations were validated by western blot (WB) and immunofluorescence (IF) using CRC cells. Immunohistochemistry (IHC) and WB using CRC patients’ tissue samples supported the relevance of the results in the real-life scenario of CRC metastasis. Finally, ELISA confirmed the association and dysregulation of GLG1 in CRC metastasis, showing GLG1 measurement in plasma of CRC patients and controls diagnostic ability of advanced stages of the disease. 

## 2. Materials and Methods

### 2.1. CRC Cell Lines

Isogenic KM12C, KM12SM, and KM12L4a CRC cell lines from I. Fidler’s laboratory (MD Anderson Cancer Center) and isogenic SW480 and SW620 CRC cell lines from the American Type Culture Collection cell repository were used [13,14].

CRC cell lines were grown at 37 °C and 5% CO_2_ in Dulbecco’s Modified Eagle Medium (DMEM, Lonza, Basel, Switzerland) supplemented with 10% fetal bovine serum (FBS, Sigma Aldrich, Saint Louis, MO, USA), 1× l-glutamine (Lonza), and 1× penicillin/streptavidin (Lonza). 

### 2.2. Protein Extracts and Quantification

Cells were grown until 90% confluence, washed three times with PBS 1×, and incubated 1 h in starving (L-glutamine and penicillin/streptavidin supplemented DMEM) to remove any trace of FBS. Then, cells were washed three times with PBS 1× and incubated for 48 h, starving at 37 °C and 5% CO_2_. After that, adherent CRC cells were harvested with PBS 1× containing 4 mM EDTA (Carl Roth, Karlsruhe, Germany), and their secretome collected. Then, 1 × 10^6^ cells per line were transferred to a new tube, centrifuged at 1200 rpm for 5 min, and the pellet was re-suspended in the corresponding buffer according to the manufacturer’s instruction (Subcellular Protein Fractionation Kit for Cultured Cells, Thermo Fisher Scientific, San Jose, CA, USA). Briefly, cells were lysed and protein extracts from five subcellular localizations were obtained (CEB, cytoplasmic proteins; MEB, membrane proteins; NEB: nuclear soluble proteins; NEB-CBP, chromatin-bound proteins; PEB, cytoskeletal proteins) [24]. Finally, proteins from the secretomes were methanol–chloroform precipitated and re-suspended in lysis buffer (RIPA, Sigma Aldrich) prior to use.

The protein concentration of the 6 subcellular fractions was obtained by the Trp quantification method [25] and confirmed by Coomassie blue staining after 10% SDS-PAGE under reduced conditions.

Paired healthy and CRC OCT-embedded frozen tissue samples from patients (*n* = 14) and CRC cells were mechanically disaggregated with 500 µL of RIPA supplemented with 1× protease and phosphatase inhibitors (MedChemExpress, Monmouth Junction, NJ, USA) until homogeneity was observed.

### 2.3. TMT 11-Plex Labeling

For the TMT 11-Plex based quantitative proteomics of the 6 subcellular fractions, 20 μg of each protein extract in a final volume of 100 μL of RIPA were reduced with 10 μL 100 mM tris(2-carboxyethyl)phosphine (TCEP, Sigma Aldrich) for 45 min at 37 °C, and 600 rpm and alkylated with 11 μL of 0.4 M chloroacetamide (Sigma Aldrich) for 30 min at room temperature (RT) and 600 rpm in darkness. Sera-Mag magnetic beads mix (50% hydrophilic/50% hydrophobic, GE Healthcare, Chicago, IL, USA) were used to improve protein digestion. Samples were incubated with 100 µL of Sera-Mag magnetic bead mix and 200 μL of acetonitrile 100% (PanReac AppliChem GmbH, Darmstadt, Germany) for 35 min at room temperature (RT) and 600 rpm to allow protein binding to the beads. Then, supernatants were discarded, and magnetic beads were washed twice with ethanol 70% (PanReac AppliChem) and acetonitrile 100%. Finally, supernatants were discarded, and proteins were joined to magnetic beads digested overnight at 37 °C and 600 rpm with 1 μg of porcine trypsin (Thermo Fisher Scientific) in 80 μL 20 mM HEPES pH 8.0 (Sigma Aldrich). The next day, samples were sonicated and supernatants were collected. All peptides from the 11 samples were separately labeled with 11 different Tandem Mass Tags (Thermo Fisher Scientific, San Jose, CA, USA, Lot UG288073), according to manufacturer’s instructions. Finally, the content of the 11 tubes was pooled together, and dried under vacuum prior to separation using a High pH Reversed-Phase Peptide Fractionation Kit (Pierce Biotechnology, Waltham, MA, USA). Desiccated samples were reconstituted in 300 μL H_2_O, TFA 0.1% (Sigma Aldrich) and digested peptides were separated into 12 fractions with the High pH Reversed-Phase columns, according to their hydrophobicity. Finally, fractions were dried under vacuum and stored at −80 °C until LC–MS/MS analysis using a Q Exactive mass spectrometer (Thermo Fisher Scientific, Bremen, Germany).

For this study, 3 different 11-Plex TMT experiments were performed to comprise the 6 subcellular fractions from the 5 different CRC cell lines employed. In addition, a pool of 20 μg of the CEB content of each CRC cell line (4 μg per CRC cell line) was included in the 3 TMT experiments and used for normalization.

### 2.4. LC–MS/MS Analysis

LC–MS/MS analyses were made according to established protocols [7,24]. In brief, an Easy-nLC 1000 nano system (Thermo Fisher Scientific, Odense, Denmark) was used for peptide separations. For each analysis, samples were loaded into a precolumn Acclaim PepMap 100 (Thermo Fisher Scientific) and eluted in a RSLC PepMap C18, 50 cm long, 75 µm inner diameter and 2 µm particle size (Thermo Fisher Scientific). The mobile phase flow rate was 300 nL/min using 0.1% formic acid in water (solvent A, Fisher Chemical, Waltham, MA, USA) and 0.1% formic acid in acetonitrile (solvent B, Fisher Chemical). The gradient profile was set as follows: 3–7% solvent B for 5 min, 7–25% solvent B for 95 min, 25–60% solvent B for 14 min, 60–95% solvent B for 1 min, and 95% solvent B for 5 min. Four microliters of each sample was injected. MS analysis was performed using a Q-Exactive mass spectrometer (Thermo Fisher Scientific).

For ionization, 1900 V of liquid junction voltage and 270 °C capillary temperature was used. The full scan method employed a *m*/*z* 300–1800 mass selection, an Orbitrap resolution of 70,000 (at *m*/*z* 200), a target automatic gain control (AGC) value of 3 × 10^6^, and maximum injection times of 100 ms. After the survey scan, the 15 most intense precursor ions were selected for MS/MS fragmentation. Fragmentation was performed with a normalized collision energy of 27 and MS/MS scans were acquired with a dynamic first mass, the AGC target was 1 × 10^5^, with a resolution of 35,000, an intensity threshold of 2 × 10^4^, an isolation window of 1.6 *m*/*z* units, and the maximum IT was 100 ms. Charge state screening was enabled to reject unassigned, singly charged, and greater than or equal to seven protonated ions. A dynamic exclusion time of 30 s was used to discriminate against previously selected ions.

### 2.5. MS Data Analysis

MS data were analyzed with MaxQuant (Version 1.6.6.0) using standardized workflows. Mass spectra *.raw files were searched against UniProt UP000005640_9606.fasta Homo sapiens (human) 2019 database (20,962 protein entries, downloaded: May 2019) using the Reporter ion MS2 type. Trypsin/P was specified as cleavage enzyme, allowing a mass tolerance of 20 ppm (Orbitrap). Precursor and reporter mass tolerance were set to 4.5 ppm and 0.003 Da, respectively, allowing 2 missed tryptic cleavages. Carbamidomethylation of cysteines was set as a fixed modification, and methionine oxidation, acetylation N-terminal, and Ser, Thr, and Tyr phosphorylation were set as variable modifications. Reporter ion intensities were bias corrected for the overlapping isotope contributions from the TMT tags according to manufacturer’s certificate. Unique and Razor peptides were considered for quantification. Minimal peptide length and maximal peptide mass were fixed to 7 amino acids and 4600 Da, respectively. Identified peptides were filtered by their precursor intensity fraction (PIF) with an FDR threshold of 0.01. Proteins identified with at least one peptide and an ion score above 99% were considered for evaluation, whereas proteins identified as potential contaminants were excluded from the analysis. The protein sequence coverage was estimated as the number of matching amino acids in a specific protein sequence that were found in the peptides sequenced having confidence ≥95% divided by the total number of amino acids in the sequence. 

To avoid differences in the amount of protein labeled by each TMT reagent, the total sum of signals of each channel were corrected by computing normalization factors to equal these sums. Sample loading (SL) normalization was carried out with R Studio (version 4.1.1) according to established protocol (https://github.com/pwilmart, accessed on 15 May 2020), using “tidyverse”, “psych”, “gridExtra” and “scales” packages (Figure S1) [26,27]. Data were then exported to Microsoft Excel 2019 and Perseus (Version 1.6.6) for the subsequent analysis. Proteins identified in at least 3 of the 10 samples with one or more peptides and a fold change ≥2 for each ratio were selected as potential proteins dysregulated in metastatic cells (upregulated = ratio ≥ 2, or downregulated = ratio ≤ 0.5). *p*-value representing the probability that the observed ratio was different than 1 by chance was also calculated. *p*-value < 0.05 was considered statistically significant. 

### 2.6. Tissue and Plasma Samples

This study related to biomarker discovery and validation was approved by the Institutional Ethical Review Boards of the Instituto de Salud Carlos III and Hospital Clínico San Carlos (Madrid) (CEI PI 13_2020-v2). Tissue and plasma samples were obtained from the IdISSC biobank of the Hospital Clínico San Carlos, which belong to the National Biobank Net (ISCIII) cofounded with FEDER funds. Written informed consent was obtained from all patients. Tissue and plasma samples were obtained according to a standardized protocol for sample collection [28,29,30]. Tissue and plasma were stored at −80 °C until use [28,29,30].

Paired OCT-embedded frozen healthy and CRC tissue from 14 CRC patients at stages I to IV was used for WB analysis (Table 1 and Table S1).

Plasma samples from healthy individuals with negative colonoscopy (*n* = 32), plasma samples from CRC patients at stages I–IV (*n* = 38), and plasma samples from individuals with premalignant lesions (colorectal adenomas; *n* = 10) were analyzed by ELISA (Table 1 and Table S2).

### 2.7. Western Blot

For WB analysis, 5 µg of each protein extract from tissue samples from patients (Table 1 and Table S1), cells, or subcellular fractions were separated on 10% SDS-PAGE under reducing conditions. Transference to nitrocellulose membranes was performed at 100 V for 90 min. Membranes blocked with 0.1% Tween PBS 1× supplemented with 3% skimmed milk were incubated with primary antibodies at optimized dilutions (Table S3) overnight (O/N) at 4 °C in the same solution. After three washes with 0.1% Tween PBS 1×, membranes were incubated with the appropriate HRP-conjugated secondary antibody (Table S3) for 1 h at RT. Membranes were then washed as above. Finally, signal developed with the ECL Pico Plus chemiluminescent reagent (Thermo Fisher Scientific) was detected on an Amersham Imager 680 (GE Healthcare, Uppsala, Sweden).

### 2.8. Immunofluorescence

Cells were fixed with 4% paraformaldehyde (Sigma Aldrich) for 20 min at 37 °C and permeabilized with PBS-0.1% Triton X-100 (Sigma Aldrich) for 10 min at room temperature and blocked with PBS-0.1% Tween 20 containing 10% FBS for 1 h at room temperature. After blocking, cells were incubated overnight at 4 °C with indicated antibodies or reagents followed by appropriate fluorophore conjugated secondary antibodies (Table S3). Cells were observed with a confocal microscope (Leica TCS SP8 AOBS UV system, Leica Microsystems, Wetzlar, Germany) after nucleus counterstaining with DAPI (1 µg/mL for 10 min at room temperature; Agilent). Images were acquired with a 63× water immersion N.A. 1.2 objective using the Leica Confocal Software (Las X). All images were acquired in the same conditions (pixel size, z-stack size, excitation laser power, and detector sensitivity). 

### 2.9. In Silico Analysis

GSEA was performed using fsgea (Version 1.16.0) and msigdbr (Version 7.2.1) R packages [31]. In brief, differential protein expression ranked lists obtained for each of the cellular compartments were subjected to pathway enrichment analysis. Pathways belonged to the gene ontology, human reactome and KEGG collections. GSEA was performed using pathways that contained at least one protein from the cross-validated differentially dysregulated proteins. From the 1831 pathways finally used in the present analysis, 1555 belonged to the gene ontology collection, 234 to the human reactome and 42 to the KEGG collection. A Benjamini–Hochberg *p* value below 0.05 was required to consider the pathway significantly enriched. String db (version 11.5) for functional protein association networks was used to identify clusters of interaction among the proteins of the dataset.

### 2.10. Immunohistochemistry, Tissue Microarrays and ELISA

For IHC analysis, previously constructed tissue microarrays (TMA) containing 95 core samples from CRC patients (Table 1) were used [32,33]. 2-μm sections were used for staining. Slides were deparaffinized as previously described [32,33,34] in low pH buffered solution (EnVision™ FLEX Target Retrieval Solution, Dako, Glostrup, Denmark). After blocking endogenous peroxidase with peroxidase blocking reagent (Dako), slides were incubated overnight at 4 °C using optimized dilutions of indicated antibodies (Table S3). To detect the antigen–antibody reaction, slides were then incubated with the appropriate anti-Ig horseradish peroxidase-conjugated polymer (EnVision™ FLEX-HRP, Dako). Visualization and immunoreactivity was conducted according to established protocols using 3,3′-diaminobenzidine as a chromogen [32,34].

GLG1 levels in plasma (1:5 diluted) were measured by ELISA (Sabbiotech, Greenbelt, MD, USA, Catalog #ABIN5594771), according to the manufacturer’s suggestions. 

### 2.11. Statistical Analysis

Microsoft Office Excel, GraphPad Prism, and the R program were used for all statistical analyses. For ELISA, variance homogeneity was evaluated with the Bartlett test. As non-homogeneous variances were observed in the dataset for all groups, the non-parametric Mann–Whitney U test was used to determine whether the mean of the control individuals, the mean of the premalignant individuals, and/or the mean of the CRC groups (grouped or analyzing each CRC stage separately) was statistically different. Mean ± SEM were represented. *p*-values < 0.05 were considered statistically significant.

ROC curves were used to evaluate GLG1 concentration as marker of CRC patients, premalignant individuals, or control individuals. Alternatively, ROC curves were used to evaluate IHC data. ROC curves were constructed with the R program (Version 3.2.3) using the R package Epi [35]. AUC and maximized sensitivity and specificity were calculated.

## 3. Results

### 3.1. Subcellular Fractionation and Analysis for Differential Protein Expression and Localization in Lymph Nodes, Liver and Lung Metastatic Tropism of Colorectal Cancer Cells

**Figure 1:**
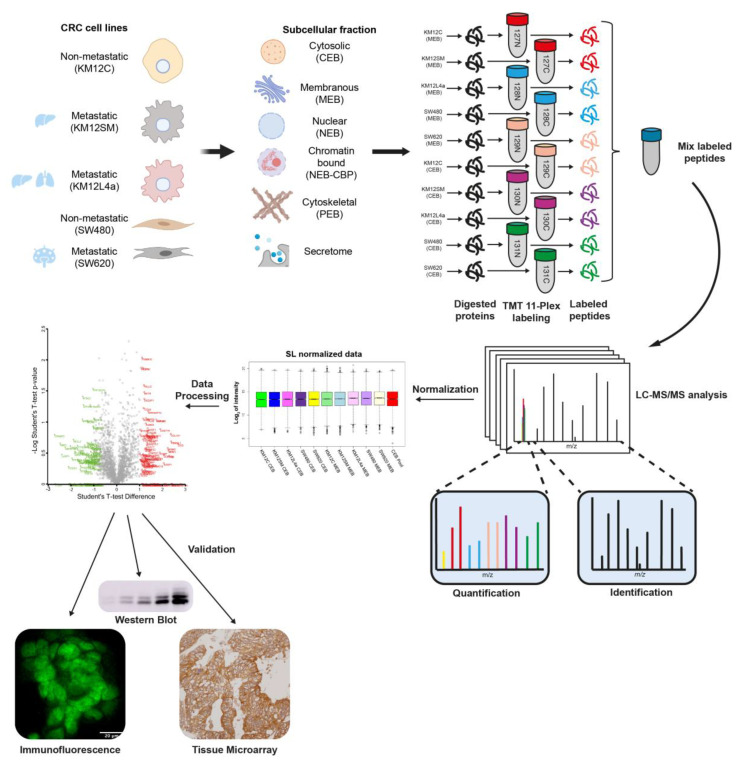
Workflow of the approach for the multidimensional proteomics analysis of the isogenic non-metastatic and metastatic colorectal cancer cell lines. CRC cells with indicated tropisms were subcellularly fractionated prior to TMT 11-plex labeling mixed in a 1:1 proportion and peptides separated using the High pH Reversed-Phase Peptide Fractionation Kit. Then, the fractionated peptides obtained per TMT experiment were analyzed onto a Q Exactive mass spectrometer. MaxQuant and Perseus were used for data analysis and identify proteins dysregulated in CRC metastasis, which were validated by different orthogonal approaches (WB, IF or IHC, among others).

Isogenic KM12 cells (non-metastatic KM12C cells, liver metastatic KM12SM cells and liver and lung metastatic KM12L4a cells) and isogenic SW480 (non-metastatic) and SW620 (lymph node metastasis) CRC cell lines with indicated metastatic tropisms were used to identify metastasis- and tropism-associated proteins (Figure 1).

Prior to in-depth proteomics analysis, cells were fractionated into five subcellular fractions (cytoplasm (CEB), membranes (MEB), nuclear proteins (NEB), chromatin-bound proteins (NEB-CBP), and cytoskeletal and insoluble proteins (PEB)). In addition to these five fractions, a sixth fraction corresponding to the conditioned medium of the cells -secretome- was also analyzed. The total of six fractions from the five cell lines were separately trypsin-digested and labeled (Figure 1). Each one of the isobaric tags of TMT 11-plex labeling kit was used to label a separate and unique fraction from an independent cell line. According to the labeling scheme used, subcellular compartments were grouped in pairs for the analysis: (i) membrane and cytoplasmic proteins, (ii) nuclear and chromatin-bound proteins, and (iii) cytoskeletal and secreted proteins. Upon labeling, the three independent TMT experiments were separately fractionated into 12 fractions according to peptide hydrophobicity prior to LC–MS/MS analysis using a Q Exactive mass spectrometer.

After normalization (Figure S1), a total of 4710 individual proteins were identified and quantified using MaxQuant, from which, 2624 proteins were observed in at least two or more pairs of subcellular compartments and 1256 proteins in all subcellular fractions (Table S4).

**Figure 2:**
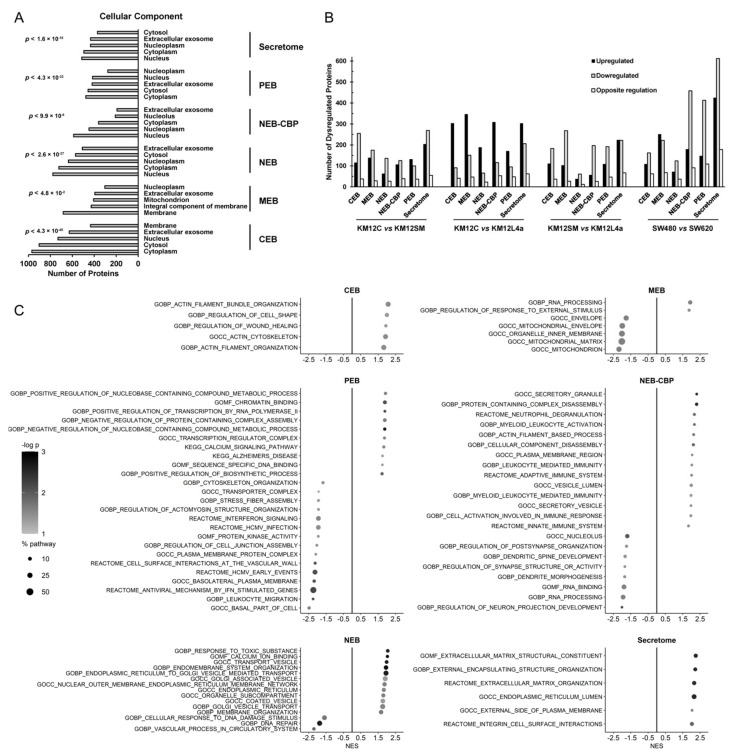
Analysis of the identified and quantified proteins in each subcellular fraction of metastatic and non-metastatic colorectal cancer cells. (**A**) Cellular component GO analysis for the subcellular classification of the proteins in each compartment was made with the DAVID database. The top five subcellular GO classifications in each fraction with the calculated *p*-value showed a good correlation with the subcellular localization of all identified and quantified proteins. (**B**) The total number of dysregulated proteins (upregulated and downregulated proteins, and those showing an opposite dysregulation in different subcellular fractions) was larger for those comparisons in which cells were more different. (**C**) Gene set enrichment analysis allowed the identification of the most dysregulated pathways in each subcellular compartment comparing metastatic vs. non-metastatic CRC cells. For each compartment and each pathway, the normalized enrichment score (NES) was calculated to identify the most dysregulated pathways according to the biological function. Positive and negative NES values denote pathway enrichment in metastatic and non-metastatic cell lines, respectively.

Next, we carried out a gene ontology (GO) cellular component classification of the identified and quantified proteins to confirm the correct subcellular fractionation of the multidimensional proteomics analysis (Figure 2A). In all of the subcellular fractions analyzed, proteins were correctly classified within the first two GO cellular component classification hits of each fraction, except for the secretome proteins, where proteins presenting dual localizations are usually found. In the secretome, 434 proteins were classified among extracellular exosome proteins.

Collectively, these results pointed out to a correct fractionation of the subcellular organelles analyzed.

### 3.2. Mapping Spatial Protein Alterations in CRC Metastatic Cells

Then, we focused the analysis on one-to-one (i.e., SW480 vs. SW620) or grouped (non-metastatic vs. metastatic) comparisons for the identification of dysregulated proteins metastasis-associated or associated with a specific tropism using Perseus (Table S5). A fold change ≥2 and ≤0.5 was used as the cut-off for upregulated and downregulated proteins, respectively. In general, we could observe a higher number of proteins downregulated than upregulated for most of the cell compartments under study. Interestingly, 582 proteins showed an opposite regulation in abundance in different compartments, indicating that these proteins were dysregulated in abundance and in localization.

The total number of dysregulated proteins for all compartments was larger for those one-to-one comparisons in which cells were more different. The total number of dysregulated proteins for the KM12C vs. KM12SM comparison was 2093, for KM12C vs. KM12L4a was 2393 and for KM12SM vs. KM12L4a, it was 2348. Furthermore, the comparison that showed the largest number of dysregulated proteins was the one between SW480 and SW620 cell lines with 3121 dysregulated proteins. These cell lines—SW480 and SW620—were arguably the two most distinct cell lines as they were both, respectively, derived from primary cells from the original tumor mass and a metastasis developed in the lymph nodes. Regarding comparisons between compartments, the subcellular compartments that showed the highest dysregulation of proteins were the secretome and nuclear and chromatin-bound proteins (Figure 2B and Figure S2).

Then, a gene set enrichment analysis (GSEA) was performed to identify the most dysregulated pathways in each subcellular compartment for grouped metastatic vs. non-metastatic CRC cells (Table S5). Taking as reference the pathways of the gene ontology, KEGG pathways and the human reactome, about 1800 altered pathways were observed using the dataset of differentially expressed proteins in each compartment (Figure 2C). For each compartment and each pathway, the normalized enrichment score (NES) was calculated. Among the top significant altered pathways, actin reorganization in CEB, RNA-processing, and response to external stimulus in MEB, vesicle mediated transport in NEB, cellular component disassembly in NEB-CBP, positive regulation of transcription and positive regulation of nucleobase containing compound metabolic process in PEB, and reorganization of extracellular matrix in secretome were observed in the analysis (Figure 2C).

Noticeably, it has been described that the secretome contains, besides exosome and extracellular proteins, other proteins that possess dual localizations in other subcellular localizations (i.e., soluble membrane receptors). Moreover, all the enriched pathways in the secretome were related to encapsulation, extracellular matrix, or integrin cell surface interactions, except reticulum or unfolded protein response that were related to extracellular protein constituents.

Collectively, these data highlight not only a correct fractionation of the subcellular fractions (including the secretome) but also a vast dysregulation of proteins and pathways in CRC isogenic cells to acquire metastatic properties. 

### 3.3. Protein clusters, Pathways and Network Analysis of Differentially Expressed Proteins in CRC Metastatic Cells

**Figure 3:**
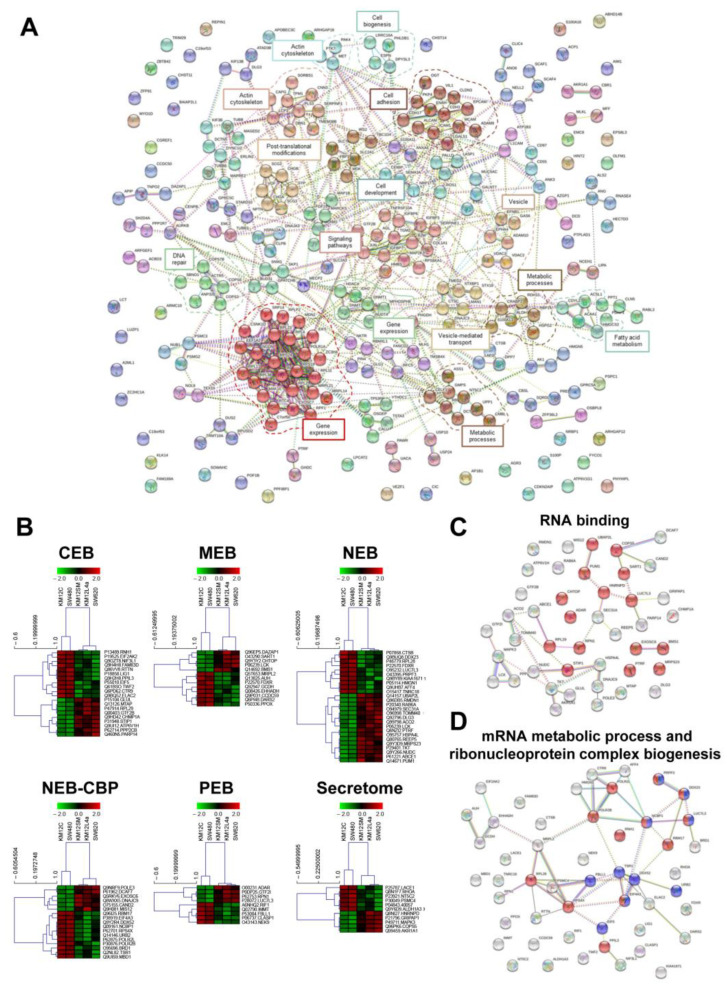
Bioinformatics analysis of the dysregulated proteins in colorectal cancer metastasis. (**A**) String revealed 15 different clusters of interaction among the dysregulated proteins associated with CRC related to DNA repair, gene expression, metabolism, signaling, cell development, cell adhesion, actin cytoskeleton, and transport or vesicle-mediated transport. (**B**) Hierarchical clustering of dysregulated—upregulated and downregulated—proteins in the different compartments showed the significant discrimination between non-metastatic (KM12C and SW480) CRC cells and metastatic (KM12SM, KM12L4a, and SW620) CRC cells (*p* < 0.05). (**C**) RNA binding (GO:0003723) in red was the molecular function more enriched in upregulated proteins in all metastatic localizations in comparison to non-metastatic cells. (**D**) mRNA metabolic process (GO:0016071) in red and ribonucleoprotein complex biogenesis (GO:0022613) in blue were the molecular functions more enriched among the downregulated proteins found to be downregulated in all metastatic localizations in comparison to non-metastatic cells.

To characterize at protein level the different metastatic properties of the cells, we carried out a global in silico analysis of the differentially-expressed proteins to visualize protein-protein interactions and the most significantly altered protein clusters and macromolecular complexes related to proteins associated with metastasis in one, two, or three of the metastatic cells lines. Among the observed vast dysregulation of proteins associated with metastasis (lymph nodes, liver, and liver and lung metastasis), released proteins together with nuclear proteins were amongst the most dysregulated proteins as assessed by String (Figure 3A), contributing to the dysregulation of different clusters of proteins related to metabolic processes, fatty acid metabolism, vesicle-mediated transport, DNA repair, cell development, signaling pathways, post-translational modifications, gene expression, and cell adhesion (*p* < 0.05). Among them, gene expression, with two different clusters containing 6 and 25 proteins (*p* < 0.05), was the most overrepresented dysregulated process.

Then, we surveyed the proteomics dataset for the identification of dysregulated proteins associated with metastasis in comparison to non-metastatic cells (Table 2 and Table S5 and Figure 3B). For such identification among the multidimensional analysis in the different subcellular organelles, proteins identified and quantified in each compartment were subjected to supervised clustering analysis (*p* value < 0.05; Kendall’s Tau algorithm) using MultiExperiment Viewer (MeV), according to their differential expression in the subcellular fractions and the metastatic properties of the cells (Figure 3B). There, the interactome of those proteins showing metastasis associated dysregulation in abundance in the different subcellular localizations was visualized. Interestingly among the proteins observed to be upregulated, we found that 15 out of the 45 commonly upregulated metastasis-associated proteins were proteins involved in RNA binding (FDR = 0.0064; Figure 3C). Regarding the downregulated proteins, we observed that the most overrepresented biological processes involved mRNA metabolic process and ribonucleoprotein complex biogenesis (FDR = 0.00011; Figure 3D).

### 3.4. Validation of Dysregulation of Proteins in Abundance and/or Localization

**Figure 4:**
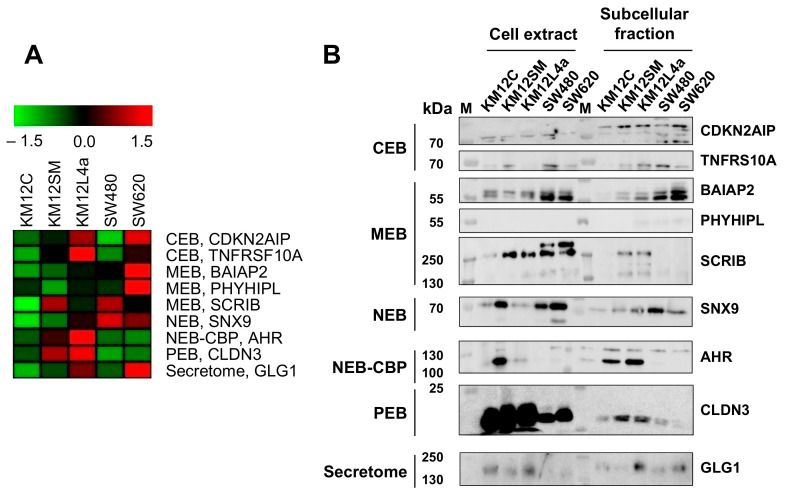
Hierarchical clustering and validation of indicated dysregulated proteins. (**A**) Unsupervised cluster analysis of the indicated altered proteins in different subcellular localizations. Green, downregulation, and red, overexpression. Color scale is related to the fold-change observed for each protein in each subcellular fraction. (**B**) A total of 10 μg of the indicated total extracts or subcellular fractions of the five isogenic CRC cells of the study were subjected to WB analysis with specific antibodies. Protein bands were quantified by densitometry.

Validation of the MS dataset and of proteins associated with metastasis was performed by meta-analysis, WB and IF. First, we observed by meta-analysis proteins previously associated with CRC metastasis, such as GAS6, MET, or MUC5AC (Table 2), which highlighted the utility of the spatial proteomics approach for the identification of CRC biomarkers [36,37]. Next, we focused the validation on selected dysregulated proteins from different subcellular compartments (Table 2). These proteins were observed upregulated in metastatic cell lines in two or more subcellular compartments or showed the highest upregulation in CRC metastatic cell lines in comparison to isogenic non-metastatic cells. According to these criteria, TNFRSF10A and CDKN2AIP from the cytoplasm, BAIAP2, PHYHIPL, and SCRIB from membrane, SNX9 from nucleus, AHR from chromatin-bound proteins, CLDN3 from the cytoskeletal fraction and GLG1 from the secretome were selected for validation (Table 2 and Figure 4A). By WB analysis, BAIAP2, SCRIB, SNX9, TNFRSF10A, PHYHIPL, CDKN2AIP, CLDN3, AHR, and GLG1 showed a concordant protein dysregulation as observed by MS (Figure 4B). A clear increment in the protein expression levels of BAIAP2, CDKN2AIP, AHR, CLDN3, and SCRIB could be observed in parallel to the metastatic properties of the cells. In addition, the differences detected at the sub-cellular level either by proteomics or by WB were in general not observed at the whole extract level, supporting the potential of the sub-cellular fractioning for elucidation of dysregulation of proteins in CRC metastasis of previously overlooked markers or highly expressed proteins. 

**Figure 5:**
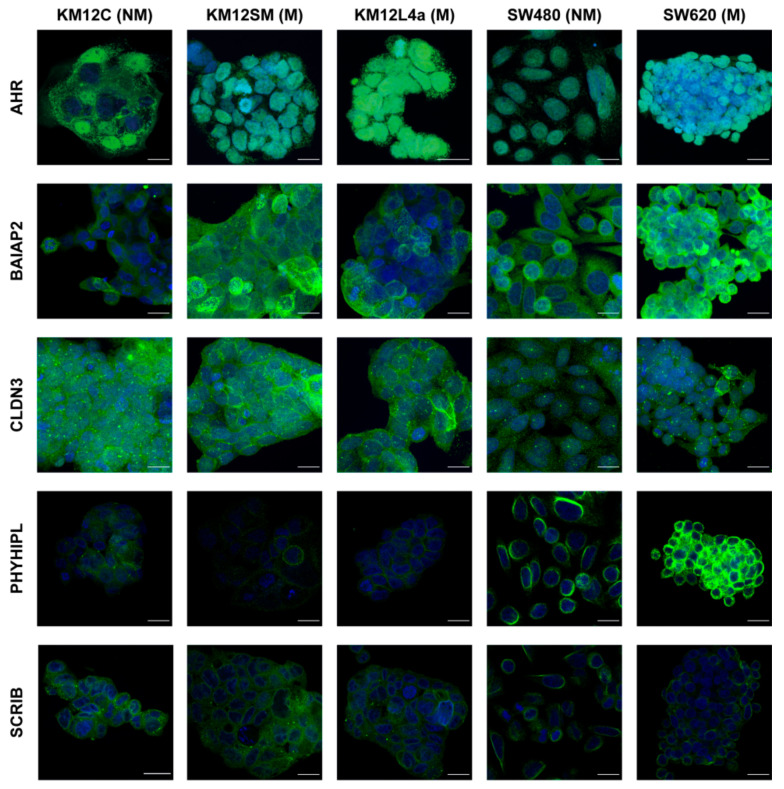
Confocal microscopy analysis of differential protein localization among KM12C, KM12SM, KM12L4a, SW480, and SW620 colorectal cancer cells. Cells were cultured on glass coverslips pretreated with Matrigel for 24 h and subjected to confocal microscopy analysis using antibodies for AHR, BAIAP2, CLDN3, PHYHIPL, and SCRIB (green). Cells were counterstained with the nuclear probe DAPI (blue). Representative images show a differential staining distribution in the different cellular compartments for these proteins between the metastatic (KM12SM, KM12L4a, and SW620 cells) and non-metastatic (KM12C and SW480) cells. M, metastatic cells. NM, non-metastatic cells. Scale Bar 20 µm. PHYHIPL and SCRIB are single stacks, whereas the other areas max projections. BAIAP2 KM12L4a cells settings were corrected to avoid saturation (see Figure S3).

In parallel, we performed IF staining of selected markers in the five CRC cells of the study under native conditions to further investigate for alterations in their subcellular localization and demonstrate their relevance in CRC (Figure 5). Besides the further confirmation that AHR, BAIAP2, CLDN3, PHYHIPL, and SCRIB are highly expressed in highly metastatic CRC cells in comparison to non-metastatic isogenic cells, changes in the localization of these proteins could also be observed between non-metastatic and metastatic cells. In this sense, we could observe differences in the localization of AHR, BAIAP2, PHYHIPL, and SCRIB and among cells. AHR shifted from a dot-like distribution, which could be associated with a vesicular distribution in the cytoplasm of KM12C cells, to a more marked nuclear localization in the metastatic KM12SM and KM12L4a cells. In the SW480 and SW620 cells, AHR was mainly located to the nucleus of the cells; although based on the fluorescence intensity, higher levels of nuclear AHR in the SW620 cells were observed in comparison to SW480. Moreover, in concordance with proteomics data, in the non-metastatic KM12C and SW480 cells, BAIAP2 was mainly localized in the cytoplasm, whereas BAIAP2 was mainly localized in the cell membrane in metastatic KM12SM, KM12L4a, and SW620 cells. PHYHIPL was localized to the cell edges and contact areas between cells in the metastatic KM12SM and KM12L4a cells, in concordance with its increase in the membrane fraction in comparison to KM12C cells previously observed by proteomics. Moreover, we could see a change in the distribution of SCRIB from being mainly located in the periphery of the nucleus to a more evenly distributed throughout the whole cell comparing SW480 and SW620 cells. Furthermore, for CLDN3, KM12L4a cells showed a more peripheral localization of the protein, while KM12C had a more even, unspecific, distribution. However, in KM12SM cells the change towards the cell edges was not as noticeable as for KM12L4a but could still be detected. Collectively, isogenic CRC metastatic cells were observed to present a vast number of changes in the abundance and in the spatial distribution of proteins that might be relevant for CRC metastasis, which would have been impossible to detect without subcellular fractionation.

### 3.5. Relevance of Dysregulated Proteins in Colorectal Cancer 

**Figure 6:**
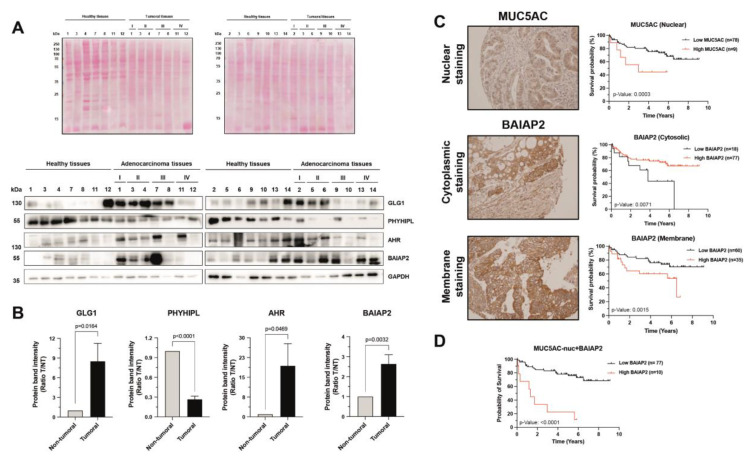
Analysis of the dysregulated proteins in CRC patients tissue samples. (**A**) Ponceau red staining and WB analysis of 14 paired tumoral and non-tumoral tissue samples from CRC patients against indicated dysregulated proteins in CRC. GLG1, AHR, and BAIAP2 were upregulated in tumoral tissue samples, with GLG1 and AHR mainly overexpressed at earlier stages of the disease. On the contrary, PHYHIPL was downregulated in tumoral tissues in comparison with paired non-tumoral tissue samples from patients. GAPDH was used as the control in the assay. Protein bands were quantified with ImageJ and normalized according to the total protein lane content of the Ponceau red staining. (**B**) GLG1, AHR, and BAIAP2, and PHYHIPL were found significantly upregulated and downregulated, respectively, in tumoral tissue samples in comparison with non-tumoral tissue samples from CRC patients. (**C**) Evaluation of the prognostic association of MUC5AC and BAIAP2 in CRC by TMA and IHC. High nuclear levels of MUC5AC and high cytoplasmic levels of BAIAP2 were significantly associated with a poor survival of CRC patients, whereas high membrane levels of BAIAP2 were associated with a higher survival of patients. (**D**) The combination of nuclear MUC5AC and membrane BAIAP2 protein staining showed the most significant differences in survival between CRC patients and healthy individuals.

Next, the selected proteins were further analyzed by WB and IHC using actual CRC samples to determine the relevance of dysregulated proteins in the disease. By WB we studied the protein content of selected proteins in paired tumoral and non-tumoral CRC patients’ tissue samples (*n* = 14) at stages I-IV. We found a statistically significant upregulation of GLG1, AHR, and BAIAP2 protein levels in tumoral tissue samples in comparison with normal tissues, with higher levels of GLG1 and AHR at early stages (stage I-II) of the disease. On the contrary, PHYHIPL was statistically found downregulated in tumoral tissues in comparison with non-tumoral tissue samples (Figure 6A,B). Noticeable, the absence of metastatic tumoral samples avoided us to confirm their dysregulation in the different sites of metastasis.

Finally, we analyzed the physiological relevance of the dysregulation of BAIAP2 by tissue microarray analysis (TMA) of patient derived samples (Figure 6C,D). MUC5AC was used as control because of its known marker character in CRC [38,39,40], and its identification and quantification in our experimental setup. MUC5AC has been previously associated with different types of cancer both as a good and bad prognostic marker [38,39,40]. Here, it was observed that nuclear levels of MUC5AC had a significant association with poor patient survival (Figure 6C). TMA analysis of BAIAP2 revealed an opposite effect for BAIAP2 depending on its localization. Patients with higher levels of BAIAP2 in the cytoplasm showed significantly better survival than those with lower levels of cytoplasmic BAIAP2. Strikingly, when looking at the membrane levels of BAIAP2, an opposite trend was observed—increased expression of BAIAP2 in the membrane led to a significant decrease in patient survival, suggesting a role for BAIAP2 in signaling when localized in the membrane (Figure 6C). Finally, we analyzed the effect of the combination of nuclear MUC5AC and membrane BAIAP2 protein levels on survival (Figure 6D). High expression of BAIAP2 in the membrane and high expression of nuclear MUC5AC showed the most significant differences in survival between patient groups.

Collectively, our results suggest that metastatic cells present changes in the spatial distribution of altered proteins mimicking actual changes in CRC tumoral samples, which were indeed associated with the prognosis of patients.

### 3.6. GLG1 Analysis as Blood-Based Candidate Biomarkers for Colorectal Cancer Diagnosis

**Figure 7:**
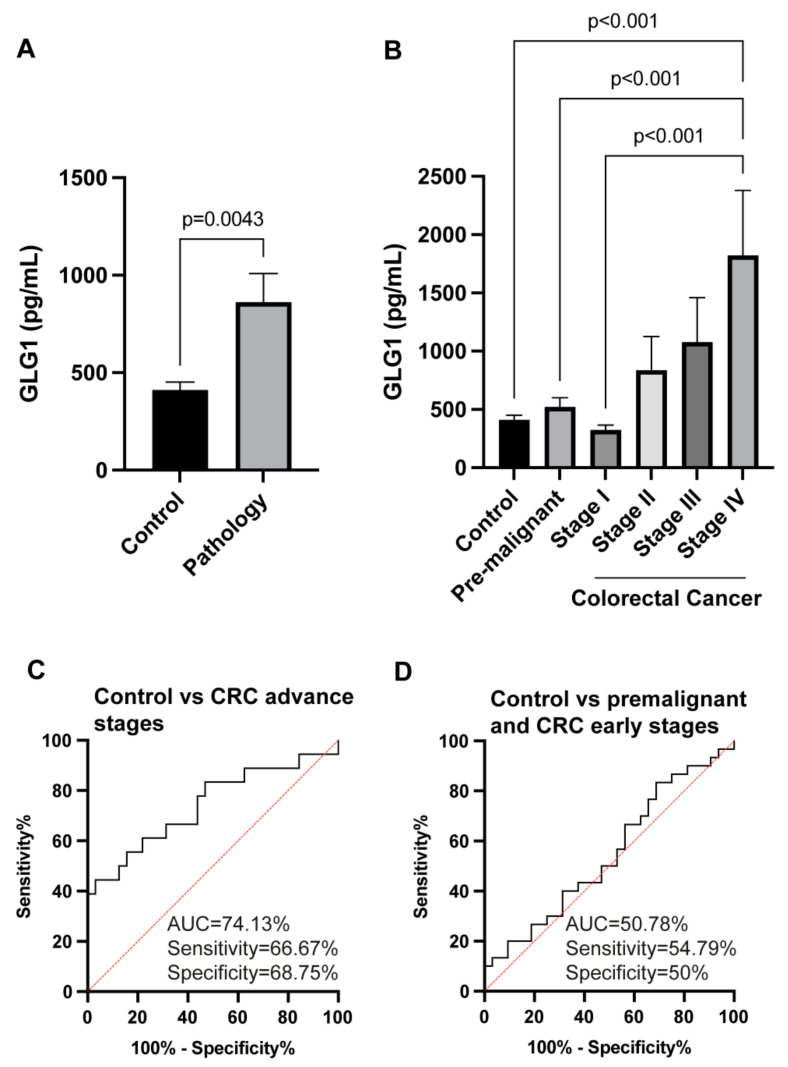
Evaluation of the plasma biomarker potential of GLG1 in colorectal cancer. (**A**) Quantification of GLG1 using commercially available ELISAs in plasma from healthy individuals as controls, and premalignant individuals and CRC patients as the pathology group. (**B**) GLG1 levels in plasma from healthy individuals as controls, premalignant individuals and stage I, II, III, and IV CRC patients. (**C**,**D**) Determination of the GLG1 value as discriminating plasma biomarker was carried out through ROC curves calculating AUC, sensitivity and specificity values. (**C**) ROC curve analysis discriminating controls and CRC patients (stages III and IV). (**D**) ROC curve analysis discriminating controls and premalignant individuals and early stage CRC patients (stages I and III).

Finally, we hypothesized that dysregulated proteins in the secretome could serve as plasma biomarkers of CRC. Thus, the levels of GLG1 in the plasma from 48 CRC patients at different stages and patients with premalignant lesions and 32 healthy individuals as controls were tested by ELISA (Figure 7). GLG1 plasma levels significantly discriminated patients from controls samples (mean ± SEM = 863.65 ± 146.21 pg/mL for the pathological group -premalignant individuals and CRC patients- versus 412.26 ± 39.82 pg/mL for controls; *p*-value = 0.0043) (Figure 7A). Importantly, GLG1 plasma levels significantly increased from premalignant individuals to stage IV CRC patients, where the highest difference in GLG1 plasma levels were observed (1822.74 ± 556.89 pg/mL for stage IV CRC patients vs. 412.26 ± 39.82 pg/mL for controls, *p*-value = 0.00017) (Figure 7B).

Finally, we surveyed the usefulness of the plasma measurement of GLG1 to discriminate CRC and premalignant individuals from controls calculating sensitivity, specificity, and AUC by means of ROC curves. The highest AUC value for GLG1 was observed for discriminating CRC stage IV from controls (*p*-value = 0.0009) with 90.63% and sensitivity and specificity of 85.71% and 78.13%, respectively. Moreover, GLG1 was observed useful for discriminating advance CRC (stages III and IV) from controls (Figure 7C), with AUC of 74.13% and sensitivity and specificity of 66.67% and 68.75%, respectively. In contrast, GLG1 was not useful and non-significant for the discrimination of CRC (stages I and II) and premalignant individuals from controls (*p*-value = 0.9251) with AUC, sensitivity and specificity of 50.78%, 54.79%, and 50%, respectively (Figure 7D). 

Collectively, these results not only confirmed the predictive value as a biomarker in plasma of GLG1 for CRC patients at advanced stages, but they also were in agreement with its higher expression in metastatic cells (as a model of advanced CRC stage) by proteomics in comparison to non-metastatic cells.

## 4. Discussion

The compartmentalization of eukaryotic cells and their distribution allows biological processes to occur synchronously requiring the specialization of multiple cellular functions. The localization of proteins to specific subcellular niches is usually a requirement to fulfil their functions and dynamics movement between compartments, which is essential for multiple cellular processes related to signaling, growth, proliferation, motility or programmed cell death. Accordingly, mislocalization of proteins has been implicated in various different pathological states, including cancer [41,42]. In this context, the exploration of the cell proteome related to metastasis in subcellular organelles or subcompartments is not only a practical approach but also a mandatory study, recognizing that proper interpretation of proteomic data requires information about compartmentation of protein machinery to get further insights into cancer metastatic processes. Therefore, determining the subcellular location of proteins and how they change in metastasis would be essential for understanding the protein’s altered biochemical functions associated with this process, comprised of invasion, and cell migration of tumoral cells from the primary tumor; intravasation and survival of tumoral cells in the circulation; extravasation, colonization, and proliferation at secondary tumor sites [6]. Moreover, the identification of specific proteins associated with metastasis or to a specific organ of colonization is a requirement to identify specific proteins as prognostic markers, altered proteins that can serve as predictive biomarkers for specific metastasis and new therapeutic targets of intervention to reduce the mortality associated with metastasis.

The combination of traditional biochemical fractionation coupled to mass spectrometry-based identification has been the next step in the characterization of the proteome subcellular organization [42]. Here, we used the most common isogenic cells in CRC as models of metastasis to lymph nodes (SW480/SW620) and the KM12 cell system (KM12C, KM12SM and KM12L4a), which mimics the metastasis to liver (KM12SM) and the metastasis to liver and lung (KM12L4a). In previous proteomics studies using KM12 isogenic cell lines, many identified proteins were described as key molecules in CRC, as VEGFA, ERBB2, EGFR, MMP7, FGFR4, cadherin-17 (CDH17), or IL13Rα2 [13,14,24,43]. In addition, in SW480 and SW620 isogenic cell lines, several proteins were also found as interesting proteins in CRC, as ITGB3, CacyBP, TFF3, or GDF15 [44,45,46]. These results highlight the necessity to continue characterizing these cell lines to increase our knowledge of CRC metastasis by quantitative proteomics analyses. In this sense, we utilized SILAC in a previous report to identify dysregulated proteins in abundance and localization between the poorly metastatic KM12C cells and the liver metastatic KM12SM CRC cells [24]. Here, our purpose of the study consisted of providing the widest quantitative analysis of multidimensional proteome alterations in CRC metastatic cells by analyzing the dysregulated proteome associated with lymph nodes, liver and lung tropisms, and their spatial distributions in the cytoplasm, membrane, nucleus, chromatin, and cytoskeletal fractions, and the secretome. This kind of high-throughput approach could provide an important way to elucidate the protein functions and to identify new roles for specific subcellular compartments. Here, this simultaneous proteomic study of different human cellular compartments provided us with novel and significant insights into protein function during differentiation and transformation of poorly metastatic cells into highly metastatic cells. Importantly, in contrast to many studies on organelle proteomics, we here provide not only a detailed list of the protein content of these organelles and the dysregulated proteins in CRC metastasis, but we also provide data validating the results of selected proteins by WB, IF, IHC, and ELISA, showing for GLG1 a remarkable association of its protein levels in plasma to late CRC stages that could be used as a predictive marker of CRC aggressiveness.

The analysis of the pathways differing between metastatic and non-metastatic CRC cells revealed that several cellular canonical pathways were over-represented among the different subcellular organelles of study. For example, the most significantly represented functions in cytoplasm (CEB) proteins were those related to actin organization and regulation of wound healing. In the membrane (MEB) fraction, RNA processing was the most enriched pathway, whereas in the nucleus the endomembrane system organization and vesicle transport appeared as enriched and DNA repair, cellular response to DNA damage, and vascular processes as diminished pathways in metastasis. Regarding cytoskeletal (PEB) proteins, the most enriched pathways were those related to metabolism, chromatin binding and regulation of transcription, whereas for the secretome upregulated proteins were associated with extracellular matrix constituents and organization and encapsulating structure organization. All of these processes are hallmarks of cancer and have been largely associated with metastasis [5]. These results and those related to the cellular component analysis pointed out to a correct subcellular fractionation of the cells.

In this work, we focused our attention on interesting proteins not previously associated with CRC or CRC metastasis and whose information in databases was scarce, such as brain-specific angiogenesis inhibitor 1-associated protein 2 (BAIAP2), Golgi apparatus protein 1 (GLG1), phytanoyl-CoA 2-hydroxylase-interacting protein-like (PHYHIPL), tumor necrosis factor receptor superfamily member 10A (TNFRSF10A), and CDKN2A-interacting protein (CDKN2AIP), in contrast to other proteins, such as SCRIB, SNX9, CLDN3, or AHR, which have been previously associated with CRC and/or CRC metastasis [47,48,49,50].

Among them, BAIAP2 has been described as being active mainly in neurons, diffused in the cytoplasm and localized in the membrane upon association to BAI1 and, thus, having a potential role in lamellipodia and filopodia formation in motile cells and as a cell adhesion molecule inducing growth cone guidance in the process of neuronal growth [51]. GLG1 has been postulated as an important regulator of membrane trafficking and described to be instrumental for metastatic colonization in bone of BM2 myeloid cells and M1a cells (derivative from the SUM159 triple negative breast cancer cell line) by binding E-selectin [52]. PHYHIPL, a paralog of the phytanoyl-CoA hydroxylase-interacting protein, is altered in glioblastoma multiforme [53], where its function remains unknown, and has been proposed as a therapeutic target gene [53]. TNFRSF10A, the receptor for the cytotoxic ligand TNFSF10/TRAIL, can regulate apoptosis mediated by TRAIL; and inactivating mutations has been demonstrated to play a role in metastasis of breast cancer [54]. CDKN2AIP was initially identified as the binding partner or ARF and several studies showed that CDKN2AIP amplification can enhance angiogenesis and its expression was closely associated with higher expression of several markers involved in angiogenesis and metastasis in breast, skin, prostate, liver and kidney cancer [55].

One of the goals of the study was to generate validated data on the here identified dysregulated proteins involved in CRC metastasis to lymph nodes, liver, and lung. To this end, we validated the results by WB, IF, IHC, and ELISA on indicated proteins from the different compartments, with and without known association to CRC metastasis. Expression levels of the selected proteins analyzed by WB were in agreement with the proteomics data. In addition, IF analysis allowed us to confirm that some of these proteins changed its localization and abundance between poorly metastatic and highly metastatic CRC cells (i.e., AHR from the cytoplasm to the nucleus, PHYHIPL from the cytoplasm to the membrane, or CLDN3 mainly from the cytoplasm to the membrane, especially in the KM12 cell system). Using actual CRC samples from patients, either by WB or IHC, it was observed that the dysregulation of GLG1, AHR, or BAIAP2 was associated with CRC (paired normal/tumoral samples) and to prognosis of CRC patients. In particular, the altered localization of BAIAP2 observed by IHC, from the cytoplasm to the membrane, was associated with a worst overall survival of CRC patients. These results validated, at least partially, the proteomics dataset pointing out to a vast dysregulation of proteins in CRC metastasis involved in processes highly related to cancer as cell biogenesis, cell adhesion, cell development, actin cytoskeleton, gene expression, signaling pathways, vesicle-mediated transport, and metabolic processes [5,56].

Finally, we demonstrated by ELISA the good performance of GLG1 as a plasma marker predictive of late stages of CRC. Our results demonstrate the usefulness of the multidimensional proteomics approach to identify dysregulated proteins in abundance and localization as novel markers in CRC. Therefore, these results support the initial premise of the study, encouraging us to perform subsequent functional analyses focused on the mechanism of action of selected dysregulated proteins to determine their relevance in the formation, progression and metastasis of CRC.

Altogether, these results demonstrate that combining or doing a whole cell analysis often dilutes protein changes altered in abundance in specific localizations that might only be observed when looking at specific subcellular fractions. Our results prove useful study protein changes within subcellular fractions to identify dysregulated proteins associated with CRC metastasis. In addition, it was possible to identify proteins whose expression in one subcellular compartment was decreased, while increased in another. Indeed, we found that about 10% of the dysregulated proteins showed opposite protein abundance dysregulation in different subcellular organelles, which would suggest regulation or activation of specific pathways involved in CRC metastasis. 

## 5. Conclusions

We provided a comprehensive proteomic analysis of CRC metastasis using isogenic CRC cell lines with different metastatic tropisms, identifying several proteins and pathways upregulated in CRC metastasis. The information gained from this study generated a large amount of data useful for determining proteins potentially involved in colon epithelial cell differentiation, transformation, and metastatic processes. Our study goes one step beyond conventional studies by providing subcellular localization of proteins associated with metastasis of CRC encompassing lymph nodes, liver and lung metastasis, completing a previous study focused only on CRC liver metastasis [24]. A further multidimensional study using actual CRC samples from patients should eventually allow for better classification and identification of dysregulated pathways, thus ensuring better diagnostics and an increased ability to provide patients with the best treatments for personalized medicine. Finally, our findings provide validated novel dysregulated proteins not-previously associated with CRC and CRC metastasis, BAIAP2, GLG1, PHYHIPL, TNFRSF10A and CDKN2AIP, which should be further explored in subsequent studies to determine their usefulness as advanced CRC stage biomarkers and should be the focus of functional experiments to determine their roles in CRC formation, progression, and metastasis, to potentially identify new therapeutic targets of the disease.

## Figures and Tables

**Table 1:** Information of the paired tumoral and non-tumoral tissue samples from CRC patients used for the WB and TMA analysis, and plasma samples from CRC patients used for the ELISA analysis.

	Application	Samples (n)		Age Average ± SD (Years)	Age Range (Years)	Gender (n)		Stage				
						Male	Female	Premalignant Individuals	I	II	III	IV
Tissue samples	WB analysis	CRC patients	14	72.1 ± 12.3	47–88	5	9	-	2	4	4	4
	IHC and TMA analysis	CRC patients	95	73.0 ± 9.6	94–51	57	38	-	3	30	61	1
Plasma samples	ELISA	CRC patients	48	73.2 ± 10.3	49–88	21	27	10	10	10	11	7
		Healthy individuals	32	57.9 ± 10.4	24–74	16	16	-				

**Table 2:** Dysregulated proteins associated with CRC metastasis (fold change ≥2 or ≤0.5) in the indicated subcellular compartment as observed in the quantitative proteomics analysis.

Protein	Accession Number	Association	Compartment	Fold Change *	Up- or Downregulated
GAS6	Q14393	Metastatic cells	Secretome	37.24	Up
DCD	P81605	Metastatic cells	Secretome	35.46	Up
c-MET	P08581	Metastatic cells	Secretome	23.31	Up
LMAN1	P49257	Metastatic cells	Secretome	19.17	Up
CEMIP	Q8WUJ3	Metastatic cells	Secretome	12.39	Up
HMGN5	P82970	Metastatic cells	PEB	11.34	Up
MUC5AC	P98088	Metastatic cells	MEB	9.85	Up
TNFRSF10A	O00220	Metastatic cells	CEB	9.37	Up
VIL1	P09327	Metastatic cells	NEB	7.71	Up
GLG1	Q92896	Metastatic cells	Secretome	7.54	Up
LCP1	P13796	Metastatic cells	CEB	6.30	Up
AGR3	Q8TD06	Metastatic cells	Secretome	5.51	Up
PLS3	P13797	Metastatic cells	Secretome	5.33	Up
CDKN2AIP	Q9NXV6	Metastatic cells	CEB	5.04	Up
SNX9	Q9Y5X1	Liver and Lung metastasis	Secretome	4.97	Up
PHYHIPL	Q96FC7	Metastatic cells	MEB	4.07	Up
ARHGAP18	Q8N392	Metastatic cells	NEB	4.01	Up
S100A16	Q96FQ6	Metastatic cells	NEB	3.98	Up
FBP1	P09467	Metastatic cells	CEB	3.96	Up
S100P	P25815	Metastatic cells	Secretome	3.30	Up
PTRF	Q6NZI2	Metastatic cells	NBP	3.02	Up
CLDN3	O15551	Liver and Liver and Lung metastasis	PEB	2.57	Up
AHR	P35869	Liver and Lung metastasis	NBP	2.52	Up
SCRIB	Q14160	Liver metastasis	MEB	2.13	Up
MAP2K3	P46734	Metastatic cells	NBP	2.10	Up
KIF13B	Q9NQT8	Metastatic cells	PEB	2.06	Up
BAIAP2	Q9UQB8	Lymph nodes metastasis	MEB	2.04	Up
LGALS1	P09382	Metastatic cells	CEB	0.20	Down
PHLDB1	Q86UU1	Metastatic cells	MEB	0.19	Down
SLC2A1	P11166	Metastatic cells	MEB	0.18	Down
FYCO1	Q9BQS8	Metastatic cells	MEB	0.16	Down
L1CAM	P32004	Metastatic cells	MEB	0.16	Down
ASS1	P00966	Metastatic cells	Secretome	0.15	Down
ICAM1	P05362	Metastatic cells	MEB	0.15	Down
MCAM	P43121	Metastatic cells	MEB	0.13	Down
TGM2	P21980	Metastatic cells	Secretome	0.05	Down
CRABP2	P29373	Metastatic cells	Secretome	0.02	Down
RBP1	P09455	Metastatic cells	Secretome	0.02	Down

*: Fold change represents the ratio metastatic/non-metastatic cells, KM12SM and KM12L4a/KM12C cells or SW620/SW480 cells.

## Data Availability

The mass spectrometry proteomics data were deposited to the ProteomeXchange Consortium via the PRIDE partner repository with the dataset identifier PXD030671 [57].

## Supplementary Materials

The following supporting information can be downloaded at: https://www.mdpi.com/article/10.3390/cells11030447/s1, Figure S1: MaxQuant data SL normalization; Figure S2: Analysis of the dysregulated proteins in CRC metastatic and non-metastatic cells; Figure S3: Confocal microscopy analysis of BAIAP2 localization between KM12C, KM12SM, KM12L4a, SW480 and SW620 CRC cells; Table S1: Information of the tumoral and non-tumoral tissue samples from CRC patients used in the study; Table S2: Plasma samples from CRC patients, individuals with premalignant lesions (polyps) and healthy individuals used from ELISA analysis; Table S3: List of antibodies used for WB, immunofluorescence and immunohistochemistry analysis; Table S4: List of proteins identified and quantified from the 11-Plex TMT experiments in KM12C, KM12SM, KM12L4a, SW480, and SW620 CRC cell lines and their expression ratios; Table S5: List of dysregulated proteins in the different subcellular fractions for all comparisons analyzed.
